# Circulating Monocytes, Tissue Macrophages, and Malaria

**DOI:** 10.1155/2019/3720838

**Published:** 2019-10-02

**Authors:** Nida Ozarslan, Joshua F. Robinson, Stephanie L. Gaw

**Affiliations:** ^1^Marmara University School of Medicine, Istanbul, Turkey; ^2^Center for Reproductive Sciences, Department of Obstetrics, Gynecology & Reproductive Sciences, University of California, San Francisco, CA, USA; ^3^Division of Maternal-Fetal Medicine, Department of Obstetrics, Gynecology & Reproductive Sciences, University of California, San Francisco, CA, USA

## Abstract

Malaria is a significant cause of global morbidity and mortality. The *Plasmodium* parasite has a complex life cycle with mosquito, liver, and blood stages. The blood stages can preferentially affect organs such as the brain and placenta. In each of these stages and organs, the parasite will encounter monocytes and tissue-specific macrophages—key cell types in the innate immune response. Interactions between the *Plasmodium* parasite and monocytes/macrophages lead to several changes at both cellular and molecular levels, such as cytokine release and receptor expression. In this review, we summarize current knowledge on the relationship between malaria and blood intervillous monocytes and tissue-specific macrophages of the liver (Kupffer cells), central nervous system (microglia), and placenta (maternal intervillous monocytes and fetal Hofbauer cells). We describe their potential roles in modulating outcomes from infection and areas for future investigation.

## 1. Introduction

Malaria is a life-threatening infectious disease of widespread burden. Globally, in 2017, 219 million people were stricken with malaria, which was an increase of 2 million cases as compared to the previous year; of these, approximately 435,000 died due to related complications [1]. Malaria was formally eliminated in the United States in 1951; however, annually ∼1,700 cases and ∼5 deaths occur per year primarily in travelers and immigrants from malaria-endemic areas. From 2000–2014, 22,029 malaria cases were reported in the United States, resulting in an economic loss of over US$500 million [2]. In Africa, where malaria is endemic, over 200 million cases of malaria occurred in 2017, accounting for approximately 92% of the total world burden [1]. Despite numerous efforts to reduce and prevent transmission, malaria remains a highly prevalent disease in the human population with devastating consequences.

*Plasmodium falciparum* is the main species affecting humans, accounting for 99.7% of malaria cases in Africa and the majority of cases in Southeast Asia, the Eastern Mediterranean, and the Western Pacific [1]. *Plasmodium vivax* is the predominant species in the Americas. Malaria is primarily transmitted by the bite of a female *Anopheles* mosquito, when sporozoites are inoculated into the human skin. They enter the peripheral circulation and then circulate in the blood until they reach the liver sinusoids to establish the liver stage. During the liver stage, sporozoites infiltrate hepatocytes and undergo multiple rounds of replication and mitotic divisions, producing a syncytial-like cell known as a schizont. Mature liver schizonts rupture, releasing merozoites into the systemic circulation which initiates the blood stage. In this stage, merozoites invade red blood cells (RBCs) and develop into mature trophozoites. Subsequently, similar to the liver stage, trophozoites rapidly replicate and divide forming the schizont. Mature schizonts synchronously rupture the infected RBCs (iRBCs) and release merozoites that eventually invade new RBCs to continue the asexual blood stage. Cyclic fever is the clinical hallmark of malaria which coincides with the synchronized release of new merozoites from iRBCs. Other classic symptoms include malaise, headache, myalgias, and nausea. Trophozoites can sequester within the vascular spaces of the central nervous system (CNS) resulting in cerebral malaria, a leading cause of death in young children, or within the intervillous spaces of placenta in pregnant women, causing placental malaria which is significantly associated with fetal growth restriction and preterm birth [3]. Depending on the degree of infection, malaria may lead to jaundice, severe anemia, hypoglycemia, acute renal failure, respiratory failure, coma, and death [4, 5].

Throughout its complex life cycle, the malaria parasite encounters the innate immune system, of which the monocyte/macrophage plays a critical role in tissue-specific inflammatory responses. In this review, we summarize current concepts on the role of cells of the monocyte/macrophage lineage in the anti-malarial response during different stages of the life cycle and their contribution to both innate and adaptive immunity. We discuss the following cell types: resident macrophages of the liver (Kupffer cells), circulating monocytes and splenic macrophages, microglial cells of the CNS, and three populations of placental macrophages (Table 1).

## 2. Dual Roles of the Monocyte/Macrophage Response

Inflammatory responses are generally categorized into two opposing functions: pro‐inflammatory and anti-inflammatory. Pro‐inflammatory responses are activated in the presence of pathogens and with the primary aim to contain and destroy them, whereas anti-inflammatory responses function to limit inflammation and resulting tissue damage, promoting wound healing for the recovery of the tissue after infection. Mediators which elicit inflammatory response pathways need to be tightly controlled since their excessive activation may have harmful effects [6], and studies have demonstrated that alterations in the balance between inflammation and immunomodulation can determine response to infection [7]. Detailed overview of this concept is beyond the scope of this review.

Monocytes/macrophages are one of the main cell types involved in the immune response. Monocytes are leukocytes originating from hematopoietic stem cells of the bone marrow. They circulate in the blood with a lifespan of 1–2 days, if not recruited to the tissue to differentiate into macrophages. Macrophages may arise from the differentiation of bone marrow-derived blood monocytes. However the majority are derived from erythromyeloid progenitors originating in the embryonic yolk sac that are long-lived and self-maintain throughout adulthood [8]. In the tissue, macrophages are involved in tissue development, homeostasis maintenance, and defense against pathogens [9]. Chronic disturbances in homeostasis may cause abnormal prolongation/augmentation of macrophage responses and lead to pathologic outcomes [10].

### 2.1. M1/M2 Polarization of Macrophages

Macrophages polarize to a spectrum of phenotypically diverse activation states in response to multiple cues but are conceptually organized into two main functional groups: the pro‐inflammatory M1 macrophage and the anti-inflammatory M2 macrophage [11]. M1 macrophages form the first line in host defense against a variety of bacteria, protozoa, and viruses, as well as in anti‐tumor immunity. In contrast, the M2 subset is characterized by diverse immunosuppressive activity and includes wound-healing macrophages, IL-10-secreting regulatory macrophages, and tumor-associated macrophages that suppress anti-tumor immunity [12]. Highly adaptive to their local microenvironment, macrophages have been shown to switch their programming from one functional phenotype to another in response to local signals and are thought to play an important role in maintaining the balance between inflammation (and associated tissue destruction) and the restoration of tissue homeostasis after infection and local injury [13].

Improper regulation of macrophage activation may lead to pathologies. For example, insulin resistance is worsened by alterations in glucose metabolism induced by M1 macrophages [14]. Cancer progression and tumor metastasis can be enhanced by M2 macrophages promoting tumor survival [10]. Along these lines, in malaria, macrophages protect the host by inducing phagocytosis, antibody-dependent cell inhibition, and cytokine production but, in some cases, may also play a part in enhancing malaria infection and associated complications [15].

## 3. Stimulation and Cytokine Response to Malaria

*Plasmodium* parasites stimulate their host by various pathways, leading to an assortment of responses on the cellular and molecular level, dependent on the degree and timing of malaria infection. The T-cell response to initial malaria infection is well described; however, other immune cells, including dendritic cells and monocyte/macrophages, have been shown to modulate immune activation and the severity of disease as well [16, 17]. For example, in mice, modest levels of *Plasmodium* infection lead to increased expression of CD40, a marker of immune activation induced by Toll-like receptors (TLRs) in dendritic cells and macrophages, which in turn enhances the expression of stimulator of interferon genes (STING), key regulators of the innate immune response pathway, and ultimately leads to augmented type I interferon (IFN) (such as IFN*α* and IFN*β*) production during early infection. These initial responses to malaria can dampen the extent of infection and related adverse effects, increasing survival rates [18]. However, the overexpression of STING can trigger a decrease in CD40 levels and inhibition of CD40-mediated inflammatory response pathways, enabling increased parasite-induced damage to the host [18].

During infection, the specificity of factors released by the different immune cell types contributes to these conflicting phenomena. The expression of certain surface molecules such as intracellular adhesion molecule-1 (ICAM-1), urokinase plasminogen activator receptor (uPAR), chemokine receptor 5 (CCR5), and CD23 was found to be elevated on monocytes during acute stage of malaria compared to convalescent infections, and large increases of ICAM-1 and uPAR were associated with severe malaria [19]. However, monocytes and macrophages do not produce typical inflammatory cytokines such as IL-6, IL-12, and TNF*α* during the earliest stages of malaria because of phagosomal acidification, whereas dendritic cells can [20]. Like in sepsis, the presence of pro‐inflammatory cytokines during acute malaria is thought to be related to the signs of severe malaria pathogenesis [21].

### 3.1. Expression of TNF and Interleukins

In malaria studies, certain receptors and cytokines are commonly investigated, such as CD163, IL-10, and TNF. Higher levels of soluble CD163 and CD25 have been found to be associated with parasitemia in general malaria patients, whereas soluble TNF receptor levels have been associated with parasitemia in pregnant patients [22]. Soluble TNF receptor levels were higher in pregnant malaria patients, especially in primigravidas (first-time mothers), and were also found to be increased after labor, presumably due normal inflammatory responses to parturition [23]. Murine studies have revealed that phagocyte-depleted mice had elevated amounts of serum TNF*α*, IL-1*β*, and IFN*γ* (a type II IFN), but not IL-10 [24]. Anti-inflammatory properties by IL-10 production are a characteristic of tissue-resident CD169^+^ macrophages [25]. Mice lacking tissue‐resident macrophages show increased malaria-related complications, such as disruptions in the blood-brain barrier, increased vascular permeability of the liver, and increased accumulation of hemozoin pigment in the lungs [25]. These studies imply a critical role for macrophages in the initial response to malaria and suggest that differential activation of these innate immune cells can modulate host response to disease.

Opsonization, the process of antibodies binding to the pathogen and marking it for ingestion and elimination by phagocytes, has also been studied in the context of malaria. It has been observed that iRBCs opsonized by immune serum increased secretion of cytokines including TNF and IL-1*β* from macrophages, whereas unopsonized iRBC only elicited the secretion of IL-6. Macrophage ingestion of these iRBCs was not required for this cytokine response [26]. On the contrary, coinfection with human immunodeficiency virus 1 (HIV-1) remarkably impaired phagocytosis and decreased IL-6 and IL-1*β* secretion by macrophages which may be the reason of unfavorable outcomes in case of HIV-1 co‐infection [27].

### 3.2. Hemozoin

Hemozoin, also called malaria pigment, is the end product of erythrocyte catabolism by *Plasmodium* parasites. Ingestion of hemozoin by monocytes/macrophages increased the secretion of IL-10, chemokine ligand 1 (CCL1), and CCL17 and expression of the mannose binding lectin receptor (CD206) [28]. The level of IL-10 secretion was found to be positively correlated with hemozoin amount in the monocytes/macrophages [29].

In summary, monocytes/macrophages are capable of varied responses to malaria in terms of cytokine secretion and receptor expression, which can be either protective or pathologic. These responses are complex, and the cross talk between the spectrum of potential responses is an important factor in determining disease outcome.

## 4. Liver Stage of Malaria and Kupffer Cells

The liver stage of malaria is the first phase of disease in the human host, resulting in the production of liver schizonts containing thousands of new merozoites that go on to establish the blood stage [30]. Kupffer cells are tissue-resident macrophages of the liver and play key roles in preventing the severity of malaria and parasite release into the blood supply. On the contrary, CD68-expressing Kupffer cells act as a major gateway through the sinusoidal barrier for the liver invasion by *Plasmodium* parasite [31]. Exposure to sporozoites does not lead to increased death of Kupffer cells; instead, marked changes in their morphology are observed [32], accompanied by increases in the levels of pro‐inflammatory (IFN*γ*, IL-2, IL-12p70, CCL20, and CCL5) and anti-inflammatory (IL-1*α*, IL-4, IL-5, IL-7, IL-17*α*, IL-13, erythropoietin, and vascular endothelial growth factor) mediators within minutes of exposure and that return to baseline within hours [33].

### 4.1. Triggering Receptor Expressed on Myeloid Cells 2 (TREM2)

The expression of TREM2, a member of the immunoglobulin superfamily that triggers phagocytosis of pathogens and cell debris, on the Kupffer cell surface has been linked to the resistance against the liver stage of malaria. In one mouse study, primary hepatocytes in direct contact with TREM2-expressing Kupffer cells had a reduced parasite burden. In contrast, expansion of *Plasmodium* parasitemia was amplified in the absence of TREM2, and Kupffer cells lacking TREM2 expression had a more anti-inflammatory polarization [34].

### 4.2. The Kupffer Cell and Apoptosis

The role of the Kupffer cell in malaria changes over the course of infection. Prior to the peak of parasitemia during the blood stage, Kupffer cells are transiently depleted and then replaced by bone-marrow-derived inflammatory monocytes which adapt to the environment and differentially express only a low number of genes [35]. After the peak of infection, Kupffer cell numbers increase and their phagocytic capacity improve [36]. Kupffer cells may act to directly limit the release of parasites into the circulation by triggering apoptotic pathways in infected hepatocytes. During sporozoite infection, Kupffer cells secrete hepatocyte growth factor (HGF) which induces apoptosis in hepatocytes of early liver schizonts, thus preventing the progression of further parasitic infection [37].

## 5. Blood Stage of Malaria, Circulating Monocytes, and Splenic Macrophages

The blood stage of malaria begins after the release of merozoites from the liver. Circulating merozoites invade RBCs and develop into trophozoite forms. Maturation of the trophozoite leads to merozoite-containing schizonts that upon rupture will reinvade new RBCs in the blood stage of *Plasmodium* infection. Humoral immunity is the main type of response against blood stage of malaria, but cell-mediated and innate immune responses also play supplementary roles in the protection of the host from parasite-induced injury [38]. These responses are mediated by circulating monocytes and splenic macrophages.

During the blood stage of malaria, the circulating monocyte population expands and increases their expression of CD1c and CD16 receptors [39]. Peripheral blood mononuclear cell (PBMC)-derived primary macrophages express increased levels of IL-6, IL-10, IL-1*β*, and TNF in malaria cases [40, 41]. In the spleen, monocyte chemoattractant protein 1 (MCP1) and MCP3 are important for macrophage maintenance as well as IL-33 which increases macrophage number and promotes the anti-inflammatory polarization of macrophages [42].

### 5.1. CD36

CD36 has an important role in the control of parasitemia during malaria by enhancing pro‐inflammatory responses and decreasing anti-inflammatory responses in the early stages of infection and increasing phagocytic activity of macrophages [43]. Absence of CD36 expression on macrophages leads to diminished TNF*α* response during the acute blood stage which results in higher parasitemia and mortality rates [44].

### 5.2. TIM Receptors and Interleukin Expression

*Plasmodium* infection increases monocyte/macrophage numbers, and these cells express decreased amounts of T-cell immunoglobulin and mucin-domain containing-3 (TIM3), an immunomodulatory molecule which leads to a decreased anti‐malarial response [45]. Blockade of TIM3 improved the phagocytosis of macrophages, and those mice produced higher levels of TNF*α*, inducible nitric oxide synthase (iNOS), and IL-12 during malaria infection [46]. Increased IL-12 production by splenic macrophages supports host resistance to the blood stage of *Plasmodium* infection in mice [47].

Infected RBCs are phagocytosed in the spleen through the interaction of phosphatidylserine expressed on iRBCs and the phosphatidylserine receptor TIM4 on splenic macrophages. During malaria, TIM4^+^ macrophage counts increase [48]. Otherwise, depletion of macrophage levels may result in increased iRBCs, resulting in endothelial sequestration and disruption of the blood flow [23, 49].

IL-23, which belongs to the IL-12 cytokine family, takes part in the protection against malaria by leading IL-17 production in splenic macrophages. IL-17 is an important cytokine for macrophage recruitment and accumulation by inducing CCL2 and CCL7 expression. Mice with lower levels of CCL2/7 had higher parasitemia and mortality rates. Thus, IL-23 production may result in better disease outcomes [50].

### 5.3. Severe Malarial Anemia

Malaria infection may lead to severe malarial anemia due to both increased iRBC rupture as well as the phagocytosis of iRBCs during which adjacent normal, uninfected RBCs are also destroyed. In one study, phagocytes engulfed collateral uninfected RBCs as compared to iRBCs at a ratio of 8 : 1 [51]. In patients with severe malarial anemia, PBMCs express elevated levels of IL-10, interferon gamma-induced protein 10 (IP10), macrophage inflammatory protein-1 beta (MIP1*β*), and MCP2 as compared to uncomplicated malaria or healthy individuals [52]. In severe malarial anemia patients, levels of CD11b, CD11c, CD18, human leukocyte antigen-DR isotype (HLA-DR), and CD86 expression and secretion of TNF*α* and IL-6 by monocytes are decreased compared to healthy controls [53]. During severe cases of malaria, type I IFN production and signaling correlate with disease outcomes, with higher levels of if type I IFN production is associated with increased anemia related mortality [54].

### 5.4. Complement System and CD35 and CD55

The complement system is an important part of the innate immune system. Complement proteins coat cells and pathogens, enhancing opsonization and phagocytosis by monocytes/macrophages. Malaria also decreases complement receptor 1 (CD35) expression in monocytes and macrophages which diminishes their phagocytosis capacity [55]. In another study, decreased expression of CD35 and complement decay accelerating factor (CD55), a molecule in the complement regulatory pathway that prevents the formation of the membrane-attack complex, was associated with the increased complement opsonization of RBCs and increased phagocytosis by macrophages. Among malaria patients with severe anemia, expression of CD35 and CD55 were significantly decreased in uninfected RBCs, whereas they were elevated in iRBCs, an interesting mechanism of immune evasion where the parasite avoids phagocytic clearance [56].

## 6. Cerebral Malaria and Microglia Cells

Cerebral malaria is the most severe acute complication of *Plasmodium* infection, a significant cause of malaria morbidity and mortality in young children. The pathology is not due to parasitic infiltration, but instead due to disruption of the blood-brain barrier and resulting hemorrhages [57]. Microglia are the self-renewing tissue-resident macrophage of the CNS parenchyma with unique embryonic origin [58, 59].

Cerebral malaria influences morphological and phenotypical changes in microglia, and increased major histocompatibility complex (MHC) class I expression was observed in activated cells [60]. Microglial activation was found to occur simultaneously with TLR4 engagement [61]. Cerebral malaria leads to an increase in monocyte/macrophage numbers, and activated microglia also induce accumulation of perivascular macrophages [60, 62]. Pathway analysis of modulated gene expression in activated microglia cells showed enrichment in cell replication and response to type I IFNs during experimental cerebral malaria. Microglia replication can be activated by type I IFNs, and direct interaction with malaria iRBC is not necessary for the activation [63]. In children with acute cerebral malaria, plasma levels of IL-2, IL-6, IL-8, IL-10, TNF*α*, and IFN*γ* were greater than controls. In the same study, all cytokine levels were higher in children who had died than the survivors [64]. These data support the idea that overexpression of pro‐inflammatory cytokines contributes to the pathogenesis of cerebral malaria.

### 6.1. IL-33

IL-33 plays an important role in the protection of experimental cerebral malaria. IL-33-treated mice had lower rates of cerebral malaria through the reduction in the early pro‐inflammatory-type response. This induces anti-inflammatory polarization of macrophages which contributes to the expansion of regulatory T cells, which in turn have a suppressive effect on helper T cells that produce the pro‐inflammatory response [42, 65].

## 7. Placental Malaria and Placental Macrophages

**Figure 1:**
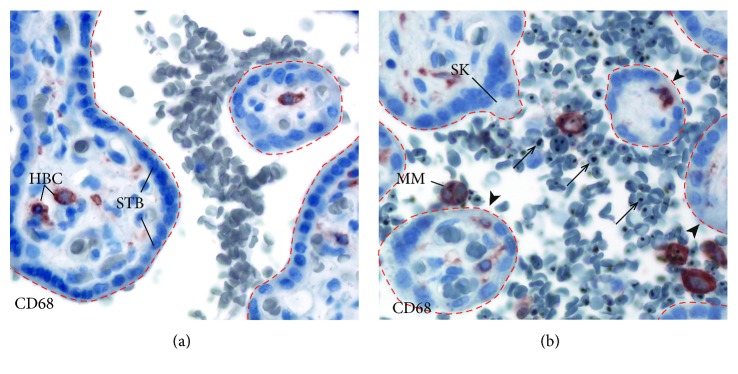
(a) Normal placenta. The dashed lines outline placental villi. Maternal RBCs are seen in the intervillous space. The placental villous (outlined with red dotted lines) is lined by the syncytiotrophoblast (STB). Fetal Hofbauer cells (HBCs) are located in the villous core. (b) Malaria-infected placenta. iRBCs (arrows) accumulate in the intervillous space, and the villous surface is denuded (arrowhead). Infected red blood cells (arrows) and maternal intervillous monocytes (MIM) are found within the intervillous space. A syncytial knot (SK) is a histologic sign of placental remodeling from pathologic processes. HBC: Hofbauer cell; STB: syncytiotrophoblast; MIM: maternal intervillous monocyte; SK: syncytial knot. Placental biopsies were stained with CD68, a monocyte/macrophage marker, and counterstained with hematoxylin.

Placental malaria is characterized by the sequestration of iRBCs in the intervillous space of the placenta (Figure 1). Approximately 25% of women living in a malaria-endemic stable transmission area have evidence of malaria infection at time of birth. Primigravity is a significant risk factor for placental malaria. Maternal outcomes of malaria during pregnancy are maternal anemia, cerebral malaria, and maternal mortality [66]. From the fetal perspective, it is a leading global cause of low birth weight, due to either fetal growth restriction and/or preterm labor. Infants born to mothers with placental malaria may develop anemia and congenital malaria [67].

Placental malaria elicits immune cell infiltration and increased cytokine production [68]. An increase in some of the inflammatory molecules in the maternal blood such as CXCL9, IL-1*β*, and IL-10 is related to increased risk of pregnancy loss or preterm delivery [3]. While the exact mechanisms remain unknown, at least three distinct macrophage subtypes may play important parts in mediating response to malaria infection in the placenta: (1) circulating monocytes arriving from the maternal spiral arteries, i.e., maternal intervillous monocytes (MIMs); (2) tissue-resident decidual macrophages of the uterus; and (3) fetal Hofbauer cells. The MIM population has been the most described in clinical pathology studies, as active placental malaria is associated with massive chronic intervillositis and placental damage [69]. Placental malaria is also associated with an increase in the fetal HBC response, and the activation state of HBCs is associated with clinical outcomes in a gravidity-dependent manner [70]. To our knowledge, there are no data on the specific role of decidual macrophages in placental malaria.

HLA-DR and CD54 are increased in MIMs in the setting of placental malaria, which correlates with hemozoin concentrations, suggesting a relative activation of monocytes in the placenta [71]. There are different findings regarding the alterations of cytokine expressions during placental malaria including increased IL-10 and IL-12 levels and decreased TNF*α* and IFN*γ* levels [72–75]. However, none of those cytokines have been found to be a reliable marker of placental malaria [76]. Whole tissue analysis of mouse placentas with and without malaria revealed increased expression of TLR4 and TLR9. In the presence of TLR4, placental malaria leads to increased production of TNF*α* which leads to a local pro‐inflammatory reaction with destructive effects [77].

### 7.1. CD163

The underlying mechanisms of macrophage activation in the placenta are an active area of research. One important molecule is CD163, a scavenger receptor specifically expressed on monocytes and macrophages. It functions in an anti-inflammatory manner through the clearance of haptoglobin-hemoglobin complexes by monocytes and macrophages which protect tissue from oxidative damage [78]. During inflammation, increased shedding of CD163 by TNF*α* cleaving enzyme leads to a rise of soluble plasma CD163. Upon *Plasmodium* infection, soluble CD163 levels are elevated. The level of soluble CD163 positively correlated with the monocyte number in the intervillous space of the placenta, whereas negatively correlated with the hemoglobin level in mother [79]. A positive correlation was found between CD163 mRNA levels and CD36 expressed by circulating monocytes, which also correlated with increased infant birth weights [80].

The placenta is a unique organ where the mother and the fetus directly interface. Of note, the mother and fetus may have conflicting reactions to infection, and the pregnancy environment promotes immune tolerance (thought to be critical in pregnancy maintenance) [81], which likely disrupts the normal immune response. While more research is needed to define the distinct roles of macrophages in mediating placental malaria. Current research findings suggest that multiple cell types respond to parasitic infection and placental injury.

## 8. Conclusion

Monocytes and macrophages are essential in the host response against malaria infections. In addition to the phagocytosis of infected cells, they also influence their surrounding tissue environment by secreting a multitude of pro‐inflammatory and anti-inflammatory mediators depending on the stage of infection and other parameters. Macrophages also respond in a diverse manner according to their tissue residency, each of which has a distinct microenvironment and respective supporting cells. However, the mechanisms modulating these responses remain largely unknown. Future studies elucidating the specific roles of tissue-resident macrophages in the response and control of stage-specific infections are needed.

## Figures and Tables

**Table 1:** Diverse cell types mediate malaria-induced immune response and injury.

Cell type	Location	Function
Kupffer cell	Liver	(i) Phagocytosis of infected RBCs (ii) Recruitment of other immune cells by secretion of cytokines (iii) Prevention of parasitic infection in hepatocytes (iv) Limiting infection through induction of apoptosis

Splenic macrophages	Spleen	(i) Secretion of cytokines
Circulating monocytes	Blood	(ii) Phagocytosis of infected RBCs

Microglia	CNS	(i) Induce accumulation of perivascular macrophages (ii) Secretion of cytokines

Hofbauer cells (fetal)	Placenta	(i) Phagocytosis of infected RBCs (ii) Secretion of cytokines (iii) Interaction between maternal/fetal immunity
Intervillous monocytes (maternal)		
Decidual macrophages (maternal)		

Description of immune cells involved in malaria response by location/origin and function. CNS, central nervous system; RBC, red blood cell.
